# Changes of central noradrenaline transporter availability in immunotherapy-naïve multiple sclerosis patients

**DOI:** 10.1038/s41598-020-70732-5

**Published:** 2020-09-04

**Authors:** Elisa Schmidt, Christian Schinke, Michael Rullmann, Julia Luthardt, Georg-Alexander Becker, Sarah Haars, Muriel Stoppe, Donald Lobsien, Karl-Titus Hoffmann, Osama Sabri, Swen Hesse, Florian Then Bergh

**Affiliations:** 1 Department of Neurology, University of Leipzig, Leipzig, Germany; 2 Department of Neurology, Charité - Universitätsmedizin Berlin, Freie Universität Berlin, Humboldt-Universität zu Berlin, Berlin Institute of Health, Berlin, Germany; 3 Department of Experimental Neurology, Charité - Universitätsmedizin Berlin, Freie Universität Berlin, Humboldt-Universität zu Berlin, Berlin Institute of Health, Berlin, Germany; 4 Berlin Institute of Health (BIH), Berlin, Germany; 5 Department of Nuclear Medicine, University of Leipzig, Leipzig, Germany; 6 Integrated Research and Treatment Center (IFB) Adiposity Diseases, University of Leipzig, Leipzig, Germany; 7 Translational Centre for Regenerative Medicine, University of Leipzig, Leipzig, Germany; 8 Department of Interventional Neuroradiology, Helios-Klinikum, Erfurt, Germany; 9 Department of Neuroradiology, University of Leipzig, Leipzig, Germany

**Keywords:** Multiple sclerosis, Molecular medicine, Multiple sclerosis, Transporters in the nervous system

## Abstract

The neurotransmitter noradrenaline (NA) mediates arousal, attention and mood, and exerts anti-inflammatory and neuroprotective effects. Alterations of monoamine signalling were reported in multiple sclerosis (MS) and psychiatric illness and may account for the high prevalence of comorbid depression and fatigue in MS patients. We assessed central noradrenaline transporter (NAT) availability using positron emission tomography (PET) and the NAT selective radiotracer S,S-[^11^C]O-methylreboxetine in immunotherapy-naïve patients with relapsing–remitting MS (RRMS; n = 11) compared to healthy controls (HC; n = 12), and its association to lesion load, time since manifestation, the expanded disability status scale (EDSS), the fatigue scale *Würzburger Erschöpfungsinventar bei MS* (WEIMuS) and Beck Depression Inventory (BDI). We found NAT availability to be increased in the thalamus, amygdala, putamen and pons/midbrain of MS patients. No relation to clinical or psychometric variables was found. These first data indicate higher NAT availability in subcortical brain regions of immunotherapy-naïve RRMS patients. If these changes of noradrenergic neurotransmission predispose to psychiatric symptoms or associate with disease activity needs to be investigated in longitudinal studies or a larger sample which allows subgroup analyses.

## Introduction

Multiple sclerosis (MS) is a chronic inflammatory immune-mediated disease of the central nervous system that is characterized by demyelination and axonal injury, predominantly affecting young adults. Besides physical disability, neuropsychological symptoms such as fatigue and depression frequently co-occur in MS patients^1–3^, leading to a substantially decreased quality of life^4^.

Autonomic and cognitive functions such as arousal, attention, drive and mood are substantially modulated by the central noradrenaline (NA) system^5^. Apart from its role as a classical neurotransmitter, NA functions as an endogenous immunomodulator that exerts anti-inflammatory and neuroprotective effects^6^. Disruptions of the central NA system have been linked to neurodegeneration in Alzheimer disease and Parkinson disease^7,8^, to the pathophysiology and symptoms of neuropsychiatric conditions such as depression^9^, anxiety^10^ and attention deficit/hyperactivity disorder^11^ and are thought to co-mediate neuro-inflammation in multiple sclerosis^12^. In vitro, NA reduces the expression of pro-inflammatory cytokines in glia cells and neurons^13,14^ and exerts neuroprotective effects by increasing the concentration of neurotrophins^15^. In experimental autoimmune encephalitis (EAE), NA levels were reported to be decreased^16^, and lesioning experiments of the locus coeruleus (as the main source of NA) led to the deterioration of clinical symptoms, while increasing central NA by NA reuptake inhibition reduced EAE severity^6^. This finding aligns with decreased NA concentrations found in the post-mortem brains of MS patients^16^, and the inverse relation of NA cerebrospinal fluid concentrations with disease duration and activity in multiple sclerosis^17^. The function of NA both in the context of immunomodulation and psychiatric symptoms may support its role as a candidate linking states of chronic neuro-inflammation and degeneration on the one hand to the comorbid psychiatric conditions frequently seen in multiple sclerosis on the other.

NAT is a critical modulator of NA signalling by limiting NA concentrations in the synaptic cleft by its reuptake into the presynaptic neuron^18^. Options to assess the activity of NA signalling in vivo in humans are limited. One method is the quantification of NA transporter (NAT) availability, using specific radiotracers and positron emission tomography (PET). There are no data on NAT availability in the living human brain of MS patients.

To assess central NAT availability in vivo, PET was applied using the highly selective radiotracer (S,S)-[^11^C]O-methylreboxetine ([^11^C]MRB)^19,20^. We expected (*i*) changes of central NAT availability in immunotherapy-naïve MS patients compared to healthy controls (HC) and explored (*ii*) whether there is an association of NAT with physical disability, time since manifestation and psychometric measures of fatigue and depressive symptoms in MS patients.

## Results

**Figure 1:**
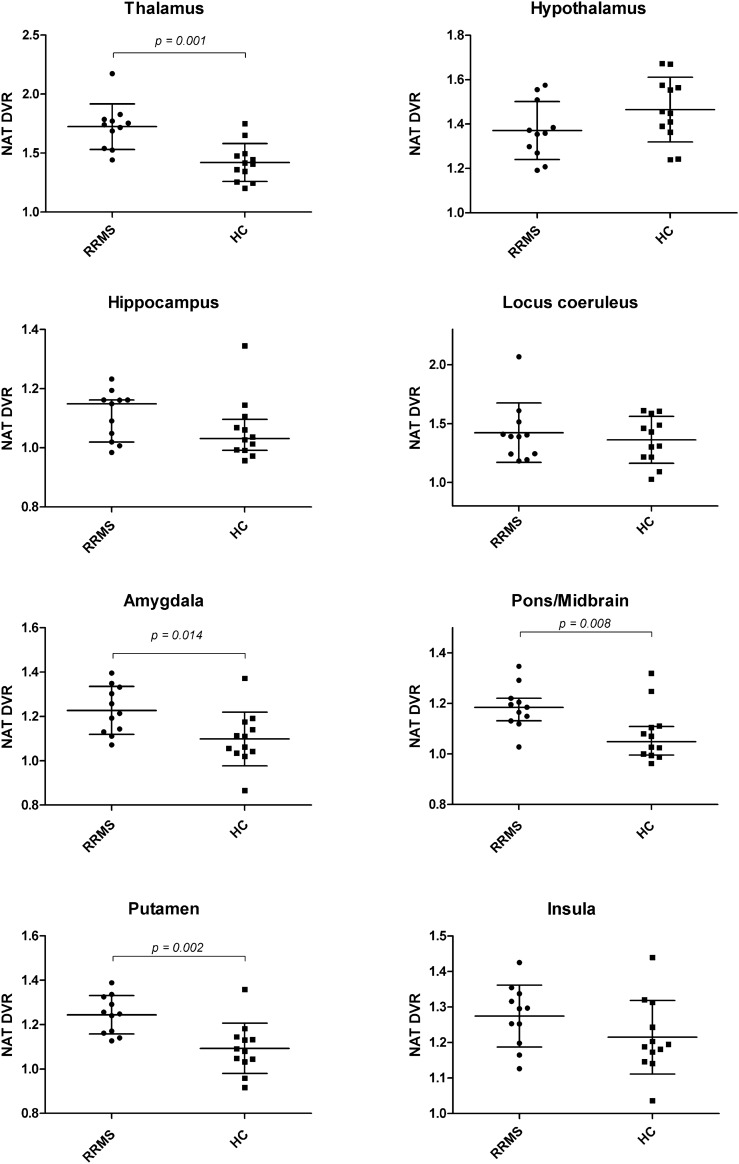
Group comparison of noradrenaline transporter (NAT) availability of patients with relapsing–remitting multiple sclerosis (RRMS; n = 11) and healthy controls (HC; n = 12). RRMS patients showed significantly higher NAT DVR in the thalamus, amygdala, pons/midbrain and putamen. Lines and whiskers indicate mean with SD for normally distributed data or, otherwise, median with interquartile range.

Subject characteristics are summarized in Table 1. Würzburger Erschöpfungsinventar bei MS scores (WEIMuS), but not the Beck Depression Inventory (BDI) were significantly higher in relapsing–remitting MS (RRMS) patients (see Table 1). Compared to HC, RRMS patients showed higher NAT Distribution Volume Ratios (DVR) in almost all subcortical regions specifically analyzed, reaching statistical significance in the thalamus (*p* = 0.001), putamen (*p* = 0.002), amygdala (*p* = 0.014) and pons/midbrain (*p* = 0.008) (Fig. 1 and Table 2). In contrast, NAT DVR was lower in the hypothalamus, although this difference was not significant (*p* = 0.12). NAT DVR of cortical regions (orbito-frontal cortex *p* = 0.87; dorsolateral prefrontal cortex *p* = 0.42; anterior cingulate cortex *p* = 0.64) and of the locus coeruleus (LC, *p* = 0.53) did not differ between the groups. Although the LC anatomically partly overlaps with the pons/midbrain region, statistical significance appears to be driven by pons/midbrain. NAT DVR were not associated with the EDSS, time since manifestation, psychometric scales or lesion volumes (see Table 3).

**Table 1:** Subject characteristics. Patients with relapsing–remitting MS (RRMS) vs. healthy controls (HC).

	*RRMS (n* = *11)*	*HC (n* = *12)*	*p*-value
Sex, male/female	4/7	6/6	0.51^c^
Age (years)	38.3 ± 9.5	33.5 ± 10.6	0.27^a^
Tracer activity (MBq)	485.6 [477.2–489.9]	380.6 [357.2–485.1]	** < 0.0001**^**b**^
BMI (kg/m^2^)	24.9 ± 3.5	23.5 ± 2.5	0.29^a^
BDI	2.0 [0–4]	1.0 [0–4.8]	0.68^b^
WEIMuS (total)	16 [8–25]	2 [0–8]	**0.004**^**b**^
WEIMuS I*/II**	5/8 [2–14 / 5–16]	0/1 [0–4 /0–5]	0.06**/0.002**^**b**^
Time since manifestation^§^ [months]	11 [6–46]		
Time since diagnosis ^§§^ [months]	4 [2–11]		
EDSS	1.5 [1.0–2.0]	-	

^a^t-test; ^b^Mann-Whitney-U test; ^c^Pearson-Chi Square test; data given as mean ± standard deviation or as median [interquartile range]. BMI, Body Mass Index; BDI, Beck Depression Inventory; WEIMuS, Würzburger Erschöpfungsinventar bei MS, *cognitive/**physical fatigue subscores, ^§, §§^ see methods section; EDSS, expanded disability status scale; **bold:** significant at p < 0.05.

**Table 2:** Distribution volume ratios (DVR) of patients with remitting-relapsing multiple sclerosis (RRMS) vs. healthy controls (HC).

	*RRMS (n* = *11)*	*HC (n* = *12)*	*p*-value
Insula	1.275 ± 0.087	1.215 ± 0.104	0.15^a^
Hippocampus	1.149 [1.020—1.162]	1.031 [0.992—1.096]	0.10^b^
Amygdala	1.227 ± 0.108	1.098 ± 0.121	**0.014**^**a**^
Nucleus accumbens	1.223 ± 0.097	1.157 ± 0.111	0.15^a^
Head of the caudate	1.124 ± 0.085	1.060 ± 0.119	0.15^a^
Putamen	1.244 ± 0.087	1.093 ± 0.113	**0.002**^**a**^
Thalamus	1.724 ± 0.194	1.420 ± 0.161	**0.001**^**a**^
Pons/midbrain	1.185 [1.131—1.220]	1.048 [0.996—1.109]	**0.008**^**b**^
Locus coeruleus	1.422 ± 0.252	1.362 ± 0.199	0.53^a^
Hypothalamus	1.371 ± 0.130	1.465 ± 0.146	0.12^a^

^a^t-test; ^b^Mann-Whitney-U test. Data given as mean ± standard deviation or as median [interquartile range].

**bold:** significant at p < 0.05.

**Table 3:** Spearman correlations of clinical parameters with NAT DVR.

	RRMS (n = 11)						
	EDSS	BDI	Weimus	Weimus I*	Weimus II**	Time since manifestation	Lesion volume (LPA)
Insula	− 0.50 (0.11)	0.26 (0.44)	0.19 (0.57)	0.39 (0.24)	0.20 (0.56)	− 0.06 (0.87)	0.10 (0.78)
Hippocampus	− 0.51 (0.11)	− 0.04 (0.91)	0.23 (0.50)	0.37 (0.27)	0.22 (0.51)	− 0.24 (0.48)	0.16 (0.64)
Amygdala	− 0.56 (0.07)	− 0.06 (0.87)	0.03 (0.94)	0.25 (0.46)	− 0.01 (0.97)	0.06 (0.87)	0.35 (0.29)
Nucleus accumbens	− 0.60 (0.05)	0.02 (0.96)	− 0.21 (0.54)	0.22 (0.51)	− 0.37 (0.26)	− 0.41 (0.20)	− 0.13 (0.71)
Head of the caudate	− 0.55 (0.08)	0.32 (0.33)	0.11 (0.75)	0.29 (0.38)	0.05 (0.88)	− 0.05 (0.89)	− 0.12 (0.73)
Putamen	− 0.26 (0.43)	− 0.15 (0.66)	0.41 (0.21)	0.38 (0.25)	0.28 (0.41)	0.08 (0.81)	0.29 (0.39)
Thalamus	− 0.16 (0.64)	0.13 (0.71)	0.18 (0.59)	0.24 (0.47)	0.22 (0.51)	0.09 (0.79)	0.27 (0.42)
Pons/midbrain	− 0.14 (0.69)	0.11 (0.75)	0.26 (0.43)	0.11 (0.76)	0.45 (0.16)	− 0.31 (0.36)	0.05 (0.89)
Locus coeruleus	− 0.45 (0.17)	0.16 (0.65)	0.46 (0.15)	0.47 (0.14)	0.28 (0.41)	− 0.06 (0.87)	− 0.26 (0.44)
Hypothalamus	− 0.21 (0.53)	− 0.13 (0.70)	− 0.21 (0.54)	− 0.13 (0.71)	0.01 (0.97)	− 0.08 (0.81)	0.11 (0.75)

Spearman-rho and p-value in parantheses. EDSS, expanded disability status scale; BDI, Beck Depression Inventory; WEIMuS, Würzburger Erschöpfungsinventar bei MS, *cognitive/**physical fatigue subscores; LPA, Lesion Prediction Algorithm; **bold:** significant at p < 0.05.

## Discussion

To the best of our knowledge, this is the first study to investigate the activity of CNS noradrenergic signalling in vivo in immunotherapy-naïve MS patients by quantifying NA transporter availability*,* and its relation to physical disability and psychometric measures of depressive symptoms and fatigue. MS patients showed higher NAT availability than HC which reached statistical significance in subcortical brain regions involving the thalamus, parts of the limbic system (amygdala, putamen) and brainstem areas (pons/midbrain). Remarkably, the only region that displayed (numerically, but non-significantly) lower availability was the hypothalamus. Whereas BDI scores were low and did not differ between the groups, the WEIMuS questionnaire revealed higher fatigue in MS patients than in healthy controls. There was no association of NAT availability with physical disability, time since manifestation, the extent of depressive symptoms, fatigue or lesion load. MS-typical lesions did not overlap with analysed brain areas and were not considered as co-variates; even if present, the small sample size would have limited the statistical power of such analysis.

Previous PET studies in multiple sclerosis focused on neuroinflammation by measuring translocator protein or adenosine receptors to detect activated microglia^21,22^, on neurodegeneration by assessing glucose and choline metabolism, on myelin content targeting myelin sheaths^23–25^, or lately, on synaptic density^26^. Our hypothesis of changes in noradrenergic signalling in MS patients was based on a wealth of data from preclinical models^6,27^, some clinical studies^28,29^ and our earlier finding of altered *serotonin* transporter availability in limbic and paralimbic regions of MS patients, which relates to psychometric measures of depression and fatigue^30^.

We found NAT availability to be *higher* in MS patients in almost all subcortical regions of interest. Dysregulation of the noradrenergic system in MS was peripherally shown by decreased NA production in peripheral blood mononuclear cells of MS patients in remission^31^ or on long-term treatment with interferon beta^32^. Centrally, NA degradation products in the cerebrospinal fluid of MS patients correlate negatively with the number of relapses and disease duration^17^, and decreased noradrenaline concentrations and locus coeruleus neuronal density were found in post-mortem brains of MS patients^16^. Taken together, these observations suggest overall *decreased* tissue NA concentrations in EAE and MS, which were attributed to an attenuated synthesis, increased metabolism or enhanced reuptake of NA^16,33^. Higher NAT availability observed in most brain areas of our study would reconcile the notion of generally lower NA tissue concentrations described in previous biochemical analyses^16^. The increased availability of the NA transporter in patients could hence reflect reduced occupancy by endogenous NA, or alternatively explain lower NA concentrations in MS by enhanced NA reuptake. In this context, the lower NAT availability in the hypothalamus merits further discussion, which stands out even though the difference did not reach statistical significance. MS is associated with hyperactivity of the hypothalamic–pituitary–adrenal (HPA) axis^34,35^, and one important driver of HPA axis activity is noradrenergic stimulation by brainstem afferents, terminating in the hypothalamus. A negative association between hypothalamic NAT availability and HPA axis responsiveness could recently be shown in other entities with pronounced stress axis activity^36^. Although we cannot support this interpretation by functional data from the same cohort, the lower hypothalamic NAT availability may reflect more pronounced NA transporter occupancy by higher synaptic concentrations of NA or lower NA reuptake in this specific target area.

NAT is located on the presynaptic membrane and parts of the axon close to the dendrites, reflecting *axonal integrity*^37^. Since neurodegeneration and axonal damage is associated with the subsequent loss of noradrenergic projections, lowered NAT availability as seen in neurodegenerative diseases such as Parkinson’s disease^38^ and Alzheimer’s disease^7^, or in the aging human brain^39^, is explained by this mechanism. Hence, decreased NAT availability, or a negative correlation between NAT availability and *physical impairment* or *time since manifestation* seemed likely. A possible explanation for the absence of these associations is the selection of MS patients at an early stage of the disease, with a low median EDSS of 1.5 and only 11 months since MS manifestation, in whom marked axonal injury and neurodegeneration are less likely^40^. Also, the EDSS measures physical abilities which are not primarily regulated by NA^41^.

We found group differences in the thalamus, putamen, amygdala and parts of the brainstem. Interestingly, an altered activation or connectivity of these brain regions was lately associated with central fatigue^42,43^ or depressive symptoms in multiple sclerosis^44^. NA as a neurotransmitter modulates drive, alertness and arousal, emotion and behaviour^5^, and the clinical efficacy of NAT inhibition in psychiatric diseases such as depression^45^, attention deficit/hyperactivity disorder^11^ as well as in MS-related depression and fatigue^46^ further supports an involvement of the noradrenergic system in the pathophysiology of these entities. Based on one study which showed higher NAT availability in the thalamus of patients with major depression^9^, a positive correlation between NAT availability and BDI scores in MS was presumable. Even though our results indicate higher thalamic NAT availability in MS, they did not reveal a relation to the BDI or fatigue scores. Similarly, Moriguchi et al. did find an association with attention, but failed to show a relation of NAT availability to the Hamilton depression score. With clinically relevant depression as an exclusion criterion, and indeed negligibly low BDI scores, our group probably lacks the symptom heterogeneity that is necessary to show such a relation. Despite higher fatigue scores in MS patients, they did not depict a relation to the NAT availability either.

Another question is if the NAT group differences we found are *region-specific*. Although it is tempting to attribute NAT changes in MS specifically to the limbic system (amygdala, striatum) or brain regions essential for the regulation of vigilance and arousal (thalamus, brainstem), it must be noted that NAT tended to be higher in almost all brain regions of the patient cohort. However, some of the analysed regions such as the hippocampus or insula have low NAT availabilities, a poorer signal-to-noise ratio and high variability^19^; hence, statistical power possibly was not sufficient to detect intergroup differences in all regions specificied in this small sample. Yet, exploratory analyses of these brain regions are of interest since they belong to noradrenergic circuits which are involved in emotion regulation and memory processing^5,47,48^.

## Conclusion

Our study indicates changes of noradrenergic signalling in immunotherapy-naïve RRMS patients. These first data require replication with larger sample sizes which allow subgroup analyses, e.g., also including patients with severe fatigue, a higher variability of depressive symptoms or cognitive deficits. Such studies might also include combined PET-MRI methods, e.g. for the assessment of neuromelanin as a marker of catecholaminergic neurons in relation to NAT availability^49^. Since the noradrenergic system is involved both in chronic inflammatory states and neuropsychiatric disorders^50^, NAT may constitute a promising candidate linking neuroinflammation with neuropsychiatric symptoms.

## Methods

### Participants

Twenty-three individuals were enrolled: eleven patients with definite relapsing–remitting MS (RRMS) according to both the criteria of Poser^51^ and the revised McDonald criteria^52^; and twelve age- and sex-matched healthy controls (HC; for subject characteristics see Table 1). RRMS patients were recruited from the outpatient clinic of the Department of Neurology, University of Leipzig. Exclusion criteria were psychiatric or neurologic disorders other than MS, a past or current history of psychopharmacotherapy, other autoimmune, chronic inflammatory or endocrine disease and hormonally active therapies (except for oral contraceptives), as well as pregnant and breastfeeding women. To screen for sub-threshold depressive symptoms, the Beck Depression Inventory (BDI) was performed at the first visit^53^. Fatigue was evaluated using the MS fatigue scale *Würzburger Erschöpfungsinventar bei MS* (WEIMuS)^54^. RRMS patients were naïve to antidepressants and disease-modifying therapy, and their most recent relapse or last treatment with corticosteroids had to be at least eight weeks before enrollment. Disease severity was rated using the Expanded Disability Status Scale (EDSS)^55^. Disease duration, or time since manifestation, is considered as the interval between the first symptoms were *reported*, in other words the first manifestation of the disease verified by residual neurological signs or correlating findings on evoked potentials or MRI, and PET data acquisition. Time since diagnosis designates the interval between the diagnosis of MS was made and PET imaging. All patients who agreed to disease modifying therapy (DMT) were treated as soon as possible after study completion. However, since we practice a system of shared decision making in the clinic, some patients decline DMT and prefer a strategy of watchful waiting. This resulted in 2 of 11 patients without treatment, who were included in the study at a routine follow-up visit. The study was conducted in accordance with the International Council for Harmonisation Guideline for Good Clinical Practice (ICH-GCP) and the declaration of Helsinki. It was approved by the local ethics committee of the Medical Faculty of the University of Leipzig (034–2007), and the German *Bundesamt für Strahlenschutz*/Federal Office for Radiation Protection (Z5-22,461/2–2013-010). All participants gave their written informed consent.

### PET imaging

PET imaging using [^11^C]MRB was performed as described previously^19^. Both the MRB standard and precursor were prepared following previously described protocols^56^. In brief, [^11^C]MRB was synthesized from [^11^C]methyliodide ([^11^C]CH_3_I) using the TRACERLab FXC automated synthesis module (GE Healthcare, USA). The final formulated product was 98% radiochemically pure, the average injected mass was 0.021 ± 0.01 μg/kg. Dynamic PET was started after intravenous bolus injection (90 s) of 485.6 [477.2 – 489.9] MBq [^11^C] MRB (MS patients) and 380.6 [357.2 – 485.1] MBq (HC; see Table 1) using the ECAT EXACT HR + scanner in 3D acquisition mode (Siemens, Erlangen, Germany; intrinsic resolution at the centre 4.3 mm [full-width at half maximum, FWHM], axial resolution: 5–6 mm, field of view: 15.5 cm). Emission scan duration was 90 min, acquiring 23 frames (4 × 0.25, 4 × 1, 5 × 2, 5 × 5, 5 × 10 min). We used a 10-min transmission scan (from three ^68^Ga sources), which was performed prior to the emission scan, for attenuation correction. An iterative reconstruction (10 iterations, 16 subsets) was applied to a transverse image series (63 slices, 128 × 128 matrix, voxel size 2.6 × 2.6 × 2.4 mm^3^) with a Hann filter (cut-off 4.9 mm) for post-processing^19^.

### Imaging data processing and analysis

**Figure 2:**
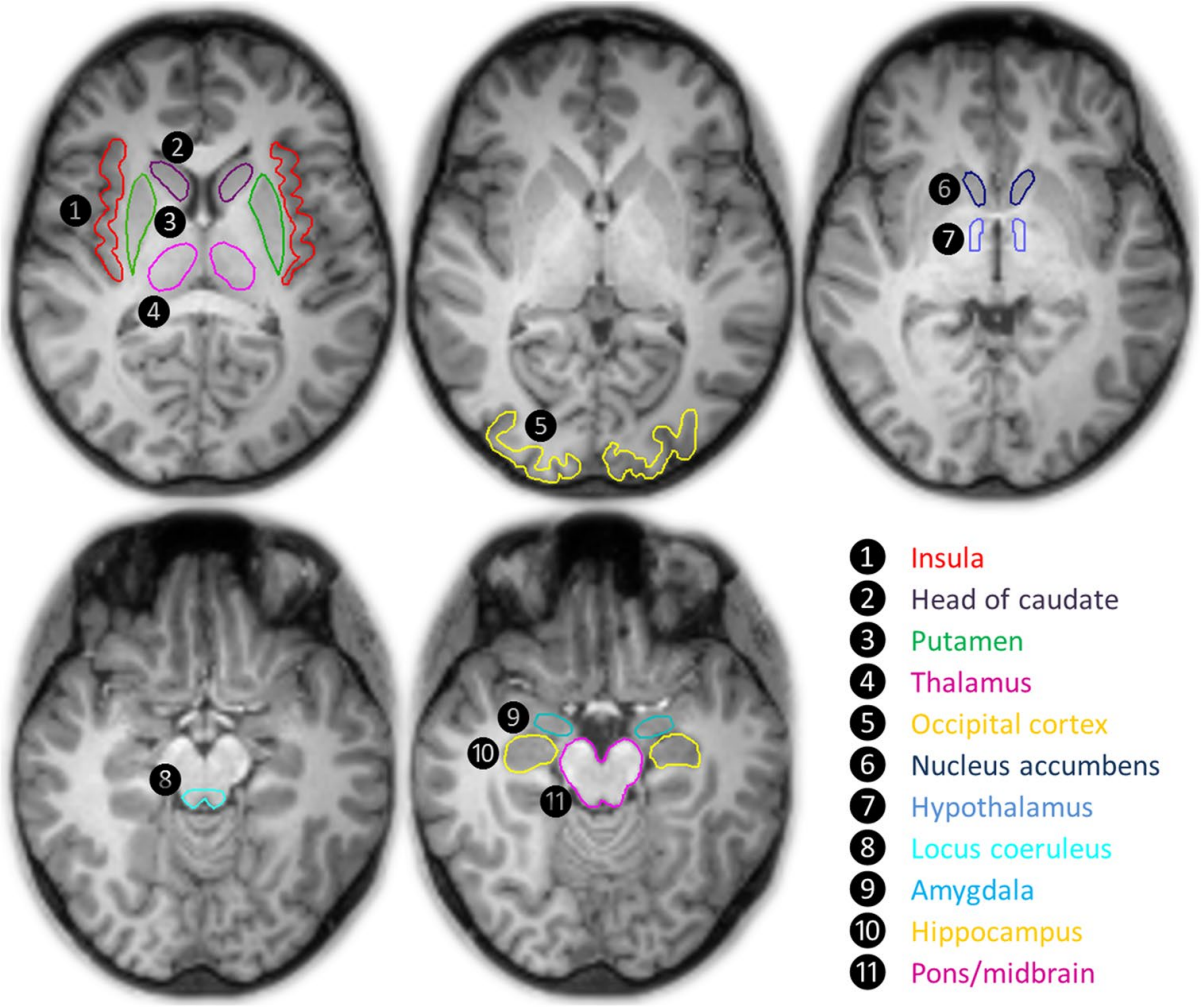
The outlined volumes of interest are exemplarily shown on an individual T1 MRI of a healthy control.

PET and MRI data analyses were performed as described previously^19^. Briefly, individual MRI data sets of the subjects were spatially reoriented (according to the anterior commissure-posterior commissure line) onto a standard brain data set similar to the Talairach space using the image processing software PMOD version 3.5 (PMOD Technologies, Zurich, Switzerland). Hereafter, volumes of interest (VOIs) were drawn manually on consecutive transverse slices of the reoriented individual MRI data sets (Fig. 2). PET data were corrected for head motion artifacts using SPM2 software (Statistical Parametric Mapping; Wellcome Trust Centre for Neuroimaging, London, UK) and then co-registered with the individual MRI data, respective of the related VOI set, to obtain via PMOD the corresponding tissue time-activity curves (TACs) from the dynamic PET data. Kinetic modelling of these regional brain TACs was performed using the multilinear reference tissue model MRTM2 (two parameters)^57^ with the occipital cortex as reference regions in PMOD (version 3.5, PMOD Technologies LLC, Zurich, Switzerland). The calculated binding potential (BP_ND_) depends linearly on the regional NAT availability and is connected to the DV ratio (DVR) of the target and the occipital cortex as the reference region by BP_ND_ = DVR-1^58,59^. The kinetic modelling yields estimates of the BP_ND_ independent of injected tracer dose and body weight. In the final computation of the DVR, the population mean value of k2′ in the thalamus from 17 study participants (described in detail in Hesse et al.^19^ ) was 0.0238 ± 0.008 1/min and used as fixed washout constant k2′_fix. MRTM2 becomes linear after a certain time, called t*^57^. For MRB, t* equals 0. Due to low tracer activity in the first 3 PET frames, we started the multilinear regression at frame 4 (0.75 min). Head movements of MS patients and HC increased at the end of the scan (after 90 or 120 min, respectively), therefore we only used time activity curves up to 90 min (frames 4 to 23) for multi-linear regression analysis. By the use of the Lesion Segmentation Tool (LST) included in Applied Statistics for SPM, we assessed lesion load as measured by the number and total volume of lesions by means of the lesion prediction algorithm (LPA)^60^. In MS patients who were investigated after HC, the injected tracer activity was increased. DVR was computed based on the reference region with linear tracer kinetics which is independent of the injected tracer activity^57^_._

### Statistical analysis

Data analyses were performed using Microsoft Excel 2010 and SPSS 23. Graphs were created using GraphPad Prism 5. All data are given as median with interquartile range (IQR) or mean ± standard deviation (SD). After the exclusion of asymmetries between corresponding brain regions using the paired t-test or Wilcoxon test, DVRs were averaged side-by-side to reduce the number of variables and multiple comparison. Data distribution was analyzed using histograms and the Shapiro–Wilk test. For data which were normally distributed, the non-paired t-test and Pearson correlation were applied for inference tests or correlative analyses. For data which were not normally distributed, the Mann–Whitney-U test or Spearman-rank correlation were applied. Two-tailed significance was applied. Results were considered significant at* p* < 0.05.

## Data Availability

The datasets used and analyzed are available from the corresponding author on reasonable request.

## Abbreviations

[^11^C]MRB: S,S-[^11^C]O-methylreboxetine
BDI: Beck depression inventory
BMI: Body mass index
BP_ND_: Binding potential
DMT: Disease modifying therapy
DRKS: German clinical trials register
DVR: Distribution volume ratio
EAE: Experimental autoimmune encephalitis
EDSS: Expanded disability status scale
FWHM: Full-width at half maximum
HC: Healthy controls
HPA: Hypothalamic–pituitary–adrenal
ICH-GCP: International council for harmonisation guideline for good clinical practice
IQR: Interquartile range
LPA: Lesion prediction algorithm
LST: Lesion segmentation tool
MRI: Magnetic resonance imaging
MRTM2: Multilinear reference tissue model (two parameters)
MS: Multiple sclerosis
NA: Noradrenaline
NAT: Noradrenaline transporter
PET: Positron emission tomography
RRMS: Relapsing–remitting MS
SD: Standard deviation
TAC: Time-activity curve
VOI: Volume of interest
WEIMuS: Würzburger Erschöpfungsinventar bei MS
